# SV40 VP1 major capsid protein in its self-assembled form allows VP1 pentamers to coat various types of artificial beads *in vitro* regardless of their sizes and shapes

**DOI:** 10.1016/j.btre.2014.12.008

**Published:** 2014-12-19

**Authors:** Masaaki Kawano, Koji Doi, Hajime Fukuda, Yoshinori Kita, Kensuke Imai, Takamasa Inoue, Teruya Enomoto, Masanori Matsui, Mamoru Hatakeyama, Yuki Yamaguchi, Hiroshi Handa

**Affiliations:** a Department of Molecular Biology, Faculty of Medicine, Saitama Medical University, 38 Morohongo, Moroyama-cho, Iruma-gun, Saitama 350-0495, Japan; b Department of Biological Information, Graduate School of Bioscience and Biotechnology, Tokyo Institute of Technology, 4259 Nagatsuta-cho, Midori-ku, Yokohama, Kanagawa 226-8501, Japan; c Department of Microbiology, Faculty of Medicine, Saitama Medical University, 38 Morohongo, Moroyama-cho, Iruma-gun, Saitama 350-0495, Japan; d Department of Nanoparticle Translational Research, Tokyo Medical University, 6-1-1 Shinjuku, Shinjuku-ku, Tokyo 160-8402, Japan

**Keywords:** SV40, simian virus 40, VLPs, virus-like particles, DTT, dithiothreitol, EGTA, ethylene glycol-bis(2-aminoethylether)-*N*,*N*,*N*′,*N*′-tetraacetic acid, EGF, epidermal growth factor, CMNPs, citrate-coated magnetic nanoparticles, RCNMV, red clover necrotic mosaic virus, NP-40, nonidet P-40, TEOS, tetraethyl orthosilicate, DMSO, dimethyl sulfoxide, PLGA, poly-lactic-*co*-glycolic-acid, DOTAP, *N*-[1-(2,3-dioleoyloxy)propyl-*N*,*N*,*N*-trimethyl ammonium chloride, W/O, water in oil, PVA, polyvinyl alcohol, W/O/W, water in oil in water, DOPE, 1,2-dioleoyl-*sn*-glocero-3-phosphoethanolamine, TEM, transmission electron microscopy, EM, electron microscopy, Polyomavirus, Simian virus 40, VP1, VP1 coating, Artificial beads, Drug delivery

## Abstract

The icosahedral capsid structure of simian virus 40 (diameter, 45 nm) consists of 72 pentameric subunits, with each subunit formed by five VP1 molecules. Electron microscopy, immuno-gold labeling, and *ζ*-potential analysis showed that purified recombinant VP1 pentamers covered polystyrene beads measuring 100, 200, and 500 nm in diameter, as well as silica beads. In addition to covering spherical beads, VP1 pentamers covered cubic magnetite beads, as well as the distorted surface structures of liposomes. These findings indicate that VP1 pentamers could coat artificial beads of various shapes and sizes larger than the natural capsid. Technology based on VP1 pentamers may be useful in providing a capsid-like surface for enclosed materials, enhancing their stability and cellular uptake for drug delivery systems.

## Highlights

• Monomeric VP1 pentamers of simian virus 40 coat artificial beads larger than natural capsids.
• Polystyrene beads (100, 200, and 500 nm in diameter) can be covered by VP1 pentamers.
• Silica beads can also be covered by VP1 pentamers.
• VP1 pentamers can also coat angular-shaped magnetite-particle and rough-shaped liposomes.
• The self-reassembly property of VP1 may allow it to coat various artificial beads, regardless of their sizes or shapes.

## 1 Introduction

Simian virus 40 (SV40) is a small non-enveloped DNA virus of the *Polyomaviridae* family. Its capsid is 45 nm in diameter and is formed by 72 pentameric subunits, each composed of five VP1 major capsid proteins (360 molecules in total). VP1 pentamers are arranged in a *T* = 7 d (triangulation number 7 dextro) icosahedral lattice, with the carboxyl terminus of VP1 mediating inter-pentameric contacts that hold the capsid together [1]. VP1 pentamers can be arranged in both five-fold and six-fold rotational positions on the icosahedral lattice of the SV40 capsid structure. Hence, the SV40 capsid is unique in that the icosahedral lattice can be formed by VP1 pentamers alone. By contrast, capsids of other viruses, such as the adenovirus capsid, are composed of pentamer and hexamer protein subunits.

When expressed in insect cells using recombinant baculovirus, VP1 self-assembles into virus-like particles (VLPs) (45 nm in diameter), similar to the natural assembly of SV40 virus capsids [2], [3]. Incubation *in vitro* of VLPs isolated from these cells with dithiothreitol (DTT) and ethylene glycol-bis(2-aminoethylether)-*N*,*N*,*N*′,*N*′-tetraacetic acid (EGTA) results in their disassembly into VP1 pentamers [4]. These VP1 pentamers can self-reassemble *in vitro* into VLPs under appropriate conditions [5], [6]. During reassembly, VLPs can encapsidate various materials, including DNA [7], [8] and proteins [9], suggesting that SV40-VLPs may be a promising platform for drug delivery systems. Additionally, VP1 may be modified to target specific cells. Foreign peptides [10] and human epidermal growth factor (EGF) [11] were inserted into VP1. Moreover, citrate-coated magnetic nanoparticles (CMNPs) were coated with VP1 pentamers, followed by conjugation of EGF protein (EGF-VP1-CMNPs) [7]. VP1 coating was found to greatly enhance the mono-dispersibility of CMNPs under physiological conditions, and in serum containing buffer, prolonging their retention in body fluids, whereas conjugation of EGF selectively guided these CMNPs onto EGF receptor expressing cells. Inclusion of EGF-VP1-CMNPs in contrast agent for magnetic resonance imaging may enhance the visualization of tumor cells overexpressing EGF receptor *in vivo*. Other VLPs can also be utilized to modify natural nanoparticles, allowing their use as contrast agents for medical imaging [12]. VLPs may also be a promising vaccine platform [13], [14]. Thus, VP1 has great potential in the development of a variety of medical nanomaterials.

Capsid subunits of various viruses were used to encapsidate artificial beads. For example, the capsid protein of a plant virus, red clover necrotic mosaic virus (RCNMV), was used to encapsidate gold nanoparticles. In that study, the surface of gold nanoparticles was immobilized with an RNA sequence to which RCNMV capsid protein binds specifically, resulting in RCNMV capsid protein being concentrated on the surface of the particles [15]. SV40 VP1 pentamers were used to encapsidate unmodified artificial beads, including quantum dot [16] and gold nanoparticles [17]. However, the importance of the structural conditions of materials encapsidated by viral capsid proteins has not been extensively studied.

This study shows that various artificial beads could be coated with VP1 pentamers, with no particular limitation of the sizes and shapes of coated artificial beads. These results indicate that VP1 coating technology is applicable to a variety of materials, providing capsid-like surface conditions and enabling high dispersibility and stability even in body fluids. Highly dispersible and stable materials coated by VP1 pentamers may be promising materials for diagnosis and treatment of various illness/diseases.

## 2 Materials and methods

### 2.1 Preparation of VP1 pentamers

VLPs consisting of wild-type VP1 were prepared as described [4]. Briefly, recombinant baculovirus expressing VP1 was generated using the baculovirus expression system (Invitrogen). Sf-9 cells were infected with these viruses, and VLPs were purified from lysates of these cells by cesium chloride density gradient. The VLPs were dialyzed in buffer containing 20 mM Tris–HCl (pH 7.9), 150 mM NaCl, and 0.1% Nonidet P-40 (NP-40), and purified VLPs were dissociated in buffer containing 20 mM Tris–HCl (pH 7.9), 150 mM NaCl, 5 mM DTT, and 5 mM EGTA. The dissociated samples were fractionated by gel filtration (Superdex 200, GE Healthcare) in the same buffer to yield monomeric VP1 pentamers.

### 2.2 Polystyrene and silica beads

Polystyrene beads (Polybead@Carboxylate 0.10 Micron Microspheres) were purchased from Polysciences, Inc. Silica beads (silica-coated CMNPs) were prepared using the seeded polymerization technique with sol–gel reaction. Briefly, a solution of tetraethyl orthosilicate (TEOS) in ethanol was added to the CMNPs in water. Silica coating was initiated by rapidly injecting an aqueous ammonia solution into the CMNP–TEOS mixture so that the water concentration of the dispersion medium was 11.0 M (1:4 (v/v) water/ethanol) and stirred overnight at 4 °C. The concentration of TEOS was varied from 2.5 to 44.1 mM, resulting in iron and ammonia concentrations of 3.6 mM and 0.9 M, respectively. Double-distilled water was added to the mixture, followed by evaporation to remove ethanol and ammonia. The resulting mixture was dialyzed against double-distilled water to obtain purified silica beads. CMNPs were prepared as described [18]. The average diameter of silica beads (110 nm) in solution was determined using ELSZ-2 (Otsuka Electronics Co., Ltd.).

### 2.3 Citric-acid-coated cubic magnetite beads

To synthesize cubic magnetite beads, the iron oleate complex was dissolved in tri-*n*-octylamine (190 g) containing oleic acid (6.14 g). The mixture was gradually heated to boiling with vigorous stirring and maintained at that temperature for 30 min. The mixture was cooled to room temperature, and a large excess of 2-propanol was added, followed by centrifugation at 7500 rpm for 1 h and redispersion in 1-octadecene. For citric-acid coating, prepared magnetite particles (180 mg) were washed with 2-propanol and suspended in toluene (16 ml). After washing, the suspension was mixed with 4 ml dimethyl sulfoxide (DMSO) containing 0.3 g of thiomalic acid and sonicated at 35 kHz for 4 h under a nitrogen atmosphere. The resulting black precipitate was washed with 2-methoxyethanol and dispersed in an aqueous solution of sodium citrate. The dispersed mixture was then sonicated at 35 kHz for 1 h. The resultant magnetite beads were precipitated by adding 1,4-dioxane and separated using a magnet. Finally, citric-acid-coated cubic magnetite beads were dispersed in distilled water and dialyzed for 3 h. The average length per side of ten randomly selected cubic magnetite beads on electron micrographs was approximately 30 nm. The average diameters of uncoated and VP1-coated cubic magnetite beads in solution were determined using ELSZ-2 (Otsuka Electronics Co., Ltd.).

### 2.4 Poly-lactic-co-glycolic-acid (PLGA) beads

Ten microliters of *N*-[1-(2,3-dioleoyloxy)propyl]-*N*,*N*,*N*-trimethyl ammonium chloride (DOTAP) in water (5 μg/ml) was added to 400 μl of PLGA in acetone (25 mg/ml) to generate a water in oil (W/O) emulsion. To this mixture was added 5 μl of 2% polyvinyl alcohol (PVA) in water under vortex to generate a water in oil in water (W/O/W) double emulsion. The latter was centrifuged at 15,000 rpm for 10 min at 4 °C, and the supernatant was removed. The resultant pellet was resuspended in 2 ml of water and stirred overnight at 4 °C to produce purified PLGA beads. The average diameter of PLGA beads (200 nm) in solution was determined using ELSZ-2 (Otsuka Electronics Co., Ltd.).

### 2.5 Preparation of cationic liposome particles

DOTAP and 1,2-dioleoyl-*sn*-glocero-3-phosphoethanolamine (DOPE) (3:1 molar ratio) were diluted in chloroform at a lipid concentration of 5 mg/ml. To prepare a lipid film, chloroform was allowed to evaporate under nitrogen flow. The film was rehydrated in Tris–HCl buffer by intermittent vortexing, and extruded through a polycarbonate filter (pore size, 100 nm) using a Mini Extruder (Avanti Polar Lipids).

### 2.6 VP1 coating of artificial beads and electron microscopy (EM)

Purified monomeric VP1 pentamers were mixed with artificial polystyrene, silica, PLGA, or citric-acid-coated cubic magnetite beads, or cationic liposomes, at a ratio of approximately 10^4^:1. Each mixture was dialyzed in coating buffer, containing 20 mM MOPS–NaOH (pH 7.0), 150 mM NaCl, and 2 mM CaCl_2_, for 16 h at room temperature. To remove free VP1 pentamers, the sample was centrifuged at 2,0400 × *g* for 15 min at 4 °C. The pellet fraction containing VP1-coated beads was washed twice and resuspended in coating buffer. For transmission electron microscopy (TEM), the samples were negatively stained with 2% ammonium molybdate and examined under a TEM (H-7500, Hitachi) as described [4].

### 2.7 Immuno-gold labeling of VP1-caoted polystyrene or silica beads

Artificial beads, either uncoated or coated with VP1 pentamers, were incubated with anti-VP1 antibody for 1 h at 37 °C, followed by incubation with protein-A-conjugated colloidal-gold (EM.PAG10, British BioCell International, Ltd.) for 1 h at 37 °C. The samples were negatively stained and examined under a TEM, as described above.

### 2.8 ζ potential of VP1-coated artificial beads

Uncoated and VP1-pentamer coated artificial beads were measured with ELSZ-2 (Otsuka Electronics Co., Ltd.) and dialyzed against buffers of pH 5, 7, and 9 using mini-dialysis units with a molecular weight cutoff of 3500 Da (Pierce, Rockford, IL).

## 3 Results

### 3.1 VP1-pentamer covered polystyrene beads

Our previous studies showed that the diameter of the particles induced by DNA-mediated self-assembly of VP1 *in vitro* was approximately 45 nm [8], and that CMNPs, 8, 20 and 27 nm in diameter, were completely encapsulated by VP1 pentamers [18], suggesting that packaging is achieved when particle diameter is under 45 nm.

**Fig. 1:**
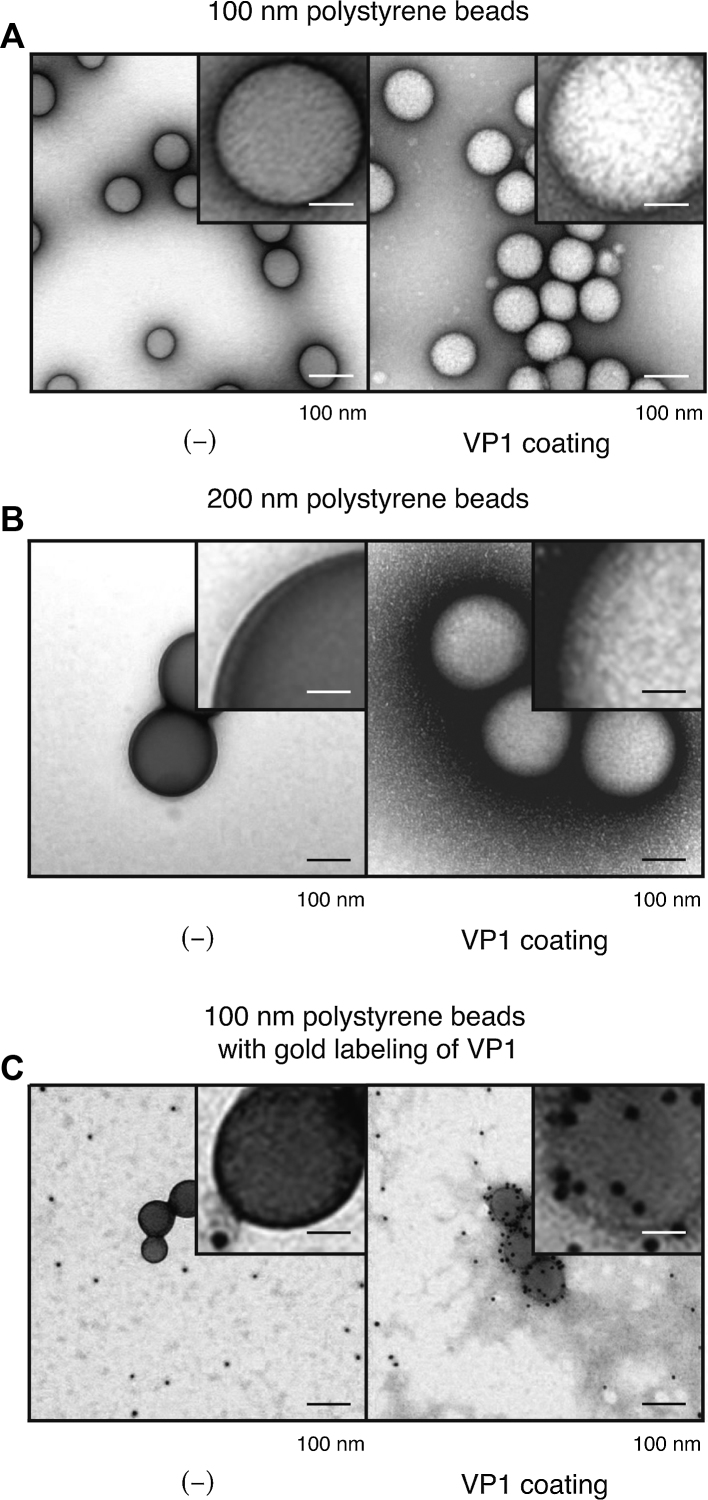
VP1 coating of polystyrene beads. A and B, polystyrene beads, (A) 100 nm and (B) 200 nm in diameter, were incubated with (right) or without (left) VP1 pentamers during dialysis. The samples were visualized by TEM with negative staining. (C) polystyrene beads incubated with (right) or without (left) VP1 pentamers were mounted on an EM grid, followed by incubation with anti-VP1 antibody and protein-A-conjugated colloidal-gold. The EM grid was observed by TEM. Scale bars: 100 nm and 25 nm (insets).

To determine the size limitations of artificial beads encapsulated by VP1 pentamers, packaging reactions were performed using polystyrene beads (100 nm and 200 nm in diameter). VP1 pentamers were mixed with polystyrene beads and dialyzed against a physiological buffer containing 20 mM MOPS–NaOH (pH 7.0), 150 mM NaCl, and 2 mM CaCl_2_ for 16 h at room temperature. After dialysis, the mixture was washed once, and a portion of the resuspended sample was mounted onto a collodion-coated copper grid and negatively stained with 2% ammonium molybdate for EM (Fig. 1A and B). Surprisingly, these polystyrene beads (100 nm and 200 nm in diameter) were covered by VP1 pentamers.

To confirm that the VP1 pentameric structure occupying the entire surface of these beads was composed of VP1 proteins, the dialyzed sample on the EM grid was incubated with anti-VP1 antibody, followed by immuno-gold labeling (Fig. 1C). As expected, gold particles accumulated on the surface of VP1-pentamer coated, but not uncoated, polystyrene beads, indicating that the polystyrene beads were covered by VP1 pentamers.

### 3.2 *ζ* potential of polystyrene beads covered by VP1 pentamers

**Fig. 2:**
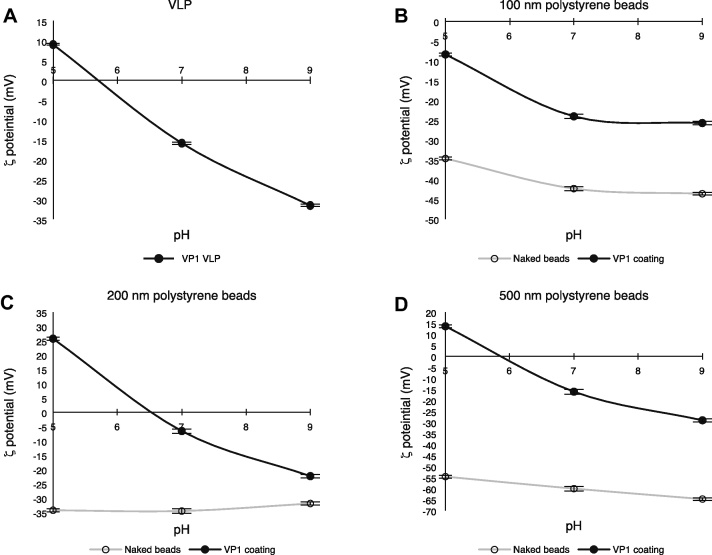
*ζ* potential of VP1-coated and uncoated polystyrene beads of various diameters. *ζ* potential of (A) wild-type VLP and (B) 100 nm, (C) 200 nm, and (D) 500 nm polystyrene beads with or without VP1 pentamers in buffers of pH 5, 7, and 9. Data are shown as the mean of the three independent experiments; error bars indicate one standard deviation.

To support this finding, the *ζ* potential of polystyrene beads before and after VP1 coating was analyzed. The *ζ* potential is the potential difference between the buffer and the stationary layer of fluid attached to the dispersed bead. Therefore, when polystyrene beads are covered with VP1 pentamers, the *ζ* potential of the beads would be moderated and closer to that of wild-type VLP. To verify this, the surface potential of VLP was assessed at pH 5, 7, and 9. This surface potential was 10 mV at pH 5, declining to −30 mV at pH 9, indicating that the electron charge on the functional groups of VP1 was altered by pH (Fig. 2A). By contrast, the surface potentials of uncoated polystyrene beads (100 nm, 200 nm, and 500 nm in diameter) ranged from −35 to −45 mV (Fig. 2B, open circles), −30 to −35 mV (Fig. 2C, open circles), and −55 to −65 mV (Fig. 2D, open circles), respectively. These findings indicated that the *ζ* potentials of the polystyrene beads were lower than that of wild-type VLP from pH 5 to pH 9. The *ζ* potential of uncoated polystyrene beads remained almost unchanged from pH 5 to pH 9 because the functional groups of these beads consist only of phenol residues, the electric charge of which is not altered in this pH range. When polystyrene beads (100 nm, 200 nm, and 500 nm in diameter) were covered with VP1 pentamers, however, their *ζ* potentials between pH 5 and pH 9 ranged from −5 to −25 mV (Fig. 2B, closed circles), 25 to −25 mV (Fig. 2C, closed circles), and 15 to −25 mV (Fig. 2D, closed circles). Thus, coating with VP1 pentamers altered the *ζ* potential of polystyrene beads, making the *ζ* potentials closer to those of VLPs. These findings also indicate that polystyrene beads as large as 500 nm in diameter can be coated with VP1 pentamers. The differences in *ζ* potentials of the three sizes of VP1-pentamer-coated polystyrene beads may be due to differences in the proportion of superficial area per unit volume of these three bead sizes.

### 3.3 VP1 coating of silica beads

**Fig. 3:**
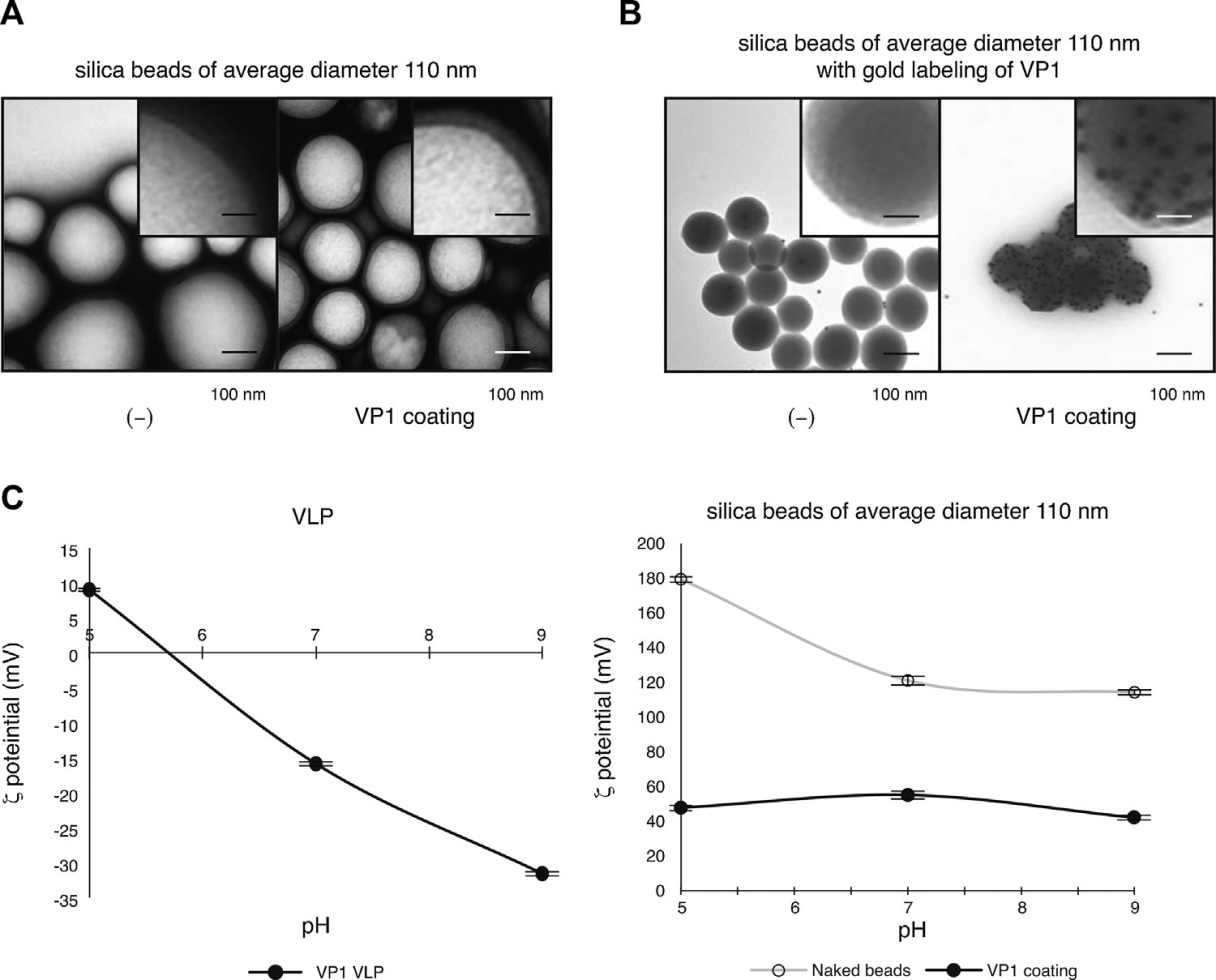
VP1 coating of silica beads. A, silica beads were incubated with (right) or without (left) VP1 pentamers during dialysis. The samples were visualized by TEM with negative staining. Scale bars: 100 nm or 25 nm (insets). B, silica beads incubated with (right) or without (left) VP1 pentamers were mounted onto EM grids and incubated with anti-VP1 antibody, followed by protein-A-conjugated colloidal-gold. The samples were visualized by TEM. Scale bars: 100 nm or 25 nm (insets). C, *ζ* potential of wild-type VLP (left) and silica beads with or without VP1 pentamers (right). Data are the mean of three independent experiments; error bars indicate one standard deviation.

To determine whether the surface conditions of artificial beads affects their coating with VP1 pentamers, the ability of these pentamers to coat silica beads was assessed. In contrast to polystyrene beads, which have phenol residues unaffected by pH as functional groups, silica beads have hydroxyl groups, the electric charge of which is altered by pH. After coating with VP1 pentamers, TEM showed the pentameric structure on the surface of silica beads (Fig. 3A). Immuno-gold labeling showed the presence of gold particles on VP1-pentamer-coated silica beads after incubation with anti-VP1 antibody (Fig. 3B).

The *ζ* potential of uncoated silica beads was 180 mV at pH 5, decreasing to 120 mV at pH 9, indicating that the electric charge of the silica side chain was altered by pH condition (Fig. 3C, right, open circles), and that this value was higher than that of VLP (Fig. 3C, left) throughout this pH range. After VP1 coating, however, the *ζ* potential of silica beads was 50 mV at pH 5, 60 mV at pH 7, and 40 mV at pH 9 (Fig. 3C, right, closed circles). Taken together, these findings show that VP1 pentamers can coat silica beads as well as polystyrene beads.

### 3.4 VP1 coating of various types of artificial beads

**Fig. 4:**
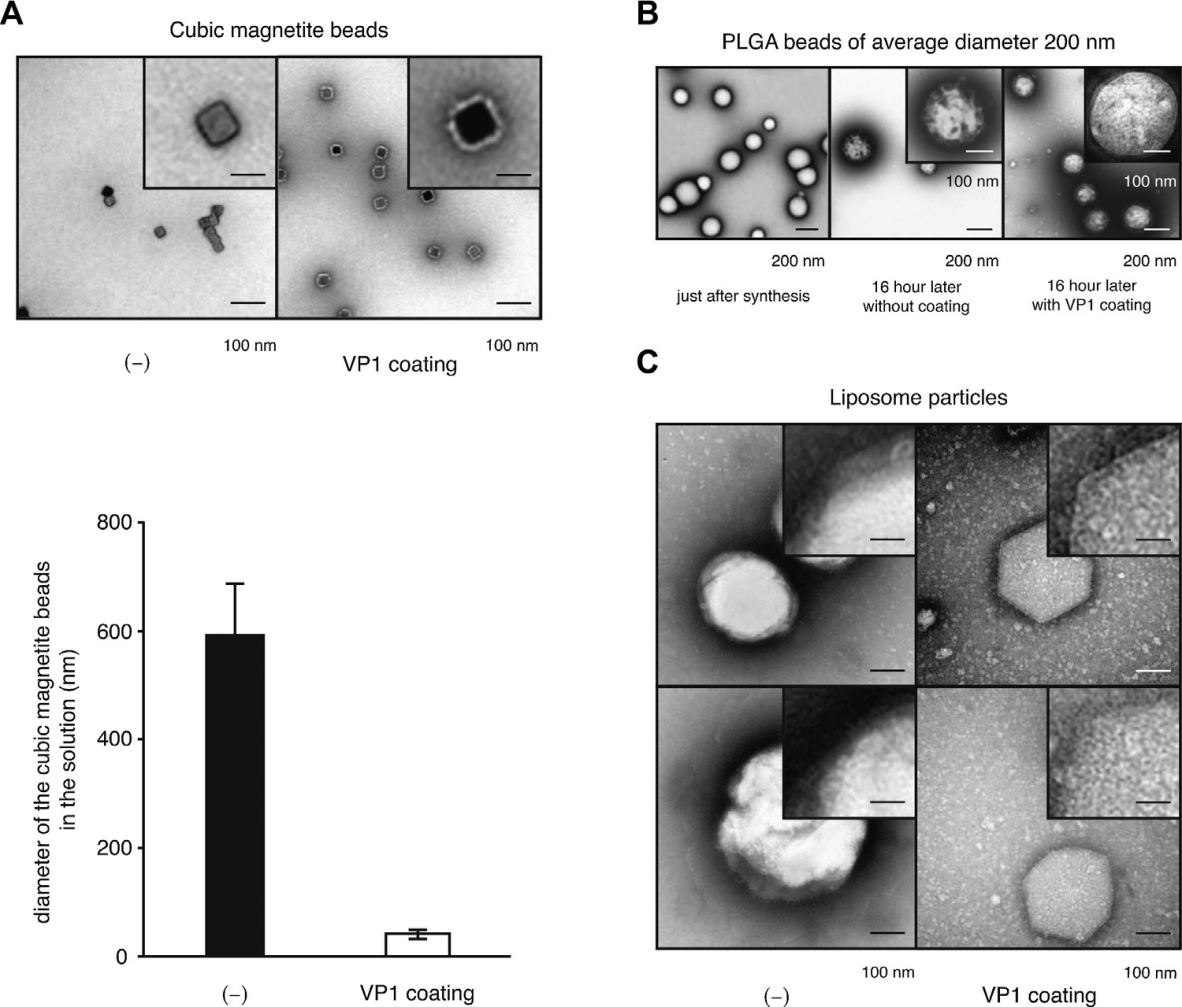
VP1 coating of various types of artificial beads. A, cubic magnetite beads were incubated with (upper right panel) or without (upper left panel) VP1 pentamers during dialysis. The samples were visualized by TEM with negative staining. Scale bars: 100 nm or 25 nm (insets). Average diameter of cubic magnetite beads in solution (lower panel). Error bars show one standard deviation. B, PLGA beads (left) were incubated with (right) or without (center) VP1 pentamers during dialysis. The samples were visualized by TEM with negative staining. Scale bars: 200 nm or 100 nm (insets). C, liposomes were incubated with (right) or without (left) VP1 pentamers during dialysis. The samples were visualized by TEM with negative staining. Scale bars: 100 nm or 25 nm (insets).

To assess the effects of bead shape on VP1 coating, various types and shapes of artificial beads were coated with VP1 pentamers. Cubic magnetite beads, biodegradable PLGA beads, and liposomes were incubated with VP1 pentamers, and their surfaces were examined by TEM (Fig. 4). Following the VP1-coating reaction, cubic magnetite beads were well-dispersed and covered with white monolayers (Fig. 4A, upper right panel) in contrast to uncoated cubic magnetite beads (Fig. 4A, upper left panel). This white monolayer was also observed on VP1-coated CMNPs [18], suggesting that the cubic magnetite beads were also likely covered by VP1 pentamers. Measurement of the average diameter in solution of cubic magnetite beads before and after VP1 coating showed that, consistent with the previous study of CMNPs [18], VP1 coating greatly enhanced the mono-dispersibility of cubic magnetite particles (Fig. 4A, lower panel), whereas uncoated cubic magnetite beads clustered in large aggregates.

Uncoated PLGA beads were spherical just after preparation (Fig. 4B, left) but formed a shriveled structure with irregular surfaces after dialysis for 16 h (Fig. 4B, center). By contrast, PLGA beads coated with VP1 pentamers maintained a spherical structure with smooth surfaces (Fig. 4B, right). In addition, TEM showed that liposomes were also covered by VP1 pentamers (Fig. 4C). These results indicate that VP1 pentamers can coat various types of artificial beads, even those with an irregular or angular structure.

## 4 Discussion

This study showed that VP1 pentamers of SV40 can coat various particles, regardless of their size, shape, and *ζ* potential. Moreover, coating or pretreatment of artificial beads was not necessary to facilitate attachment of VP1 pentamers. To elucidate the surface conditions required for VP1 coating, the ability of VP1 pentamers to coat two types of artificial beads was assessed: polystyrene beads with lower potential and silica beads with higher potential. Surprisingly, EM showed that both types of beads were coated with VP1 pentamers. Moreover, the *ζ* potential of coated larger sized beads was close to that of 45 nm VLP, indicating that VP1 pentamers covered these beads. In addition to polystyrene and silica beads, VP1 pentamers were capable of coating cubic magnetite and rough-surfaced liposomes.

Immobilization of a viral RNA sequence onto the artificial beads was found to be necessary for bead coating by a capsid protein of RCNMV [15]. VP1-pentamer coating, however, did not require specific bead modifications [16]. Moreover, SV40 VP1 pentamers could coat artificial beads larger than wild-type SV40 capsid.

VP1 pentamers may be able to self-reassemble, such that purified VP1 pentamers could automatically reassemble into VLPs without the need of any cellular factors [19]. In the presence of double-stranded DNA, purified VP1 pentamers formed a capsid structure 45 nm in diameter [8]. During this process, VP1 pentamers attached to DNA molecules, followed by construction of the encapsidating icosahedral capsid structure. Furthermore, CMNPs (8, 10, and 27 nm in diameter) were coated by self-reassembling VP1 pentamers [18]. The C-terminal region of VP1 was found to be critical for the complete coverage of CMNPs by VP1 pentamers because loss of this region resulted in the nonspecific attachment of VP1 pentamers to CMNPs and incomplete coverage of CMNPs, as well as an inability of VP1 pentamers to form the capsid structure [20], [21]. Based on these results, we hypothesize that self-assembly of VP1 pentamers through their C-terminal region plays an important role in VP1 coating of the materials described in the current study.

One of the reasons that SV40 VP1 pentamers successfully coated various materials in this study was likely due to the use of gel-filtered monomeric VP1 pentamers. Gel filtration was found to yield two VP1 peaks, corresponding to the void volume (>670 kDa) and approximately 200 kDa [2], [4], [5], indicating that VP1 pentamers are present as both multimeric and monomeric forms after dissociation in the presence of EGTA and DTT. In this study, gel-filtered dissociated VP1 pentamers were used to coat various particles. According to the quasi equivalence theory for the development of icosahedral lattices [22], any icosahedral symmetric structures can be constructed with five-fold pentameric units and six-fold hexameric units. For example, a triangulation number 1 icosahedral lattice consists of 12 five-fold pentameric units; a triangulation number 3 lattice consists of 12 five-fold pentameric units plus 20 six-fold hexameric units; and a *T* = 7 d icosahedral lattice, such as the SV40 capsid, consists of 12 five-fold pentameric units plus 60 six-fold hexameric units. Crystallography of the SV40 capsid shows that VP1 pentamers are present as both five-fold pentameric and six-fold hexameric units in the icosahedral lattice [1], [23]. Since monomeric VP1 pentamers can be present in both five-fold and six-fold positions, the SV40 icosahedral capsid is formed by VP1 pentamers alone. One of the benefits of utilizing monomeric VP1 pentamers may be their ability to form both five-fold pentameric units and six-fold pentameric units on the icosahedral lattice. Therefore, we hypothesize that monomeric VP1 pentamers could be located at any position on artificial beads, even when forming icosahedral lattices larger than the wild-type SV40 capsid. In addition to VP1 coating of the larger artificial beads, cubic beads and rough-shaped liposome beads could also be coated by monomeric VP1 pentamers, suggesting that these pentamers are sufficiently flexible to coat various shaped artificial beads by adjusting the spacing and curvature between these pentamers, depending on the surface structure of the artificial beads. VP1-coated cubic magnetite beads retained their cubic structure, indicating that the VP1 pentamers adjusted their positioning. Thus, VP1-pentamer coating of liposomes may alter them from amorphous rounded particles to regular faceted particles on fluid surface of liposomes. Although VP1 pentamers may adjust their coordination on artificial beads, the interactions between VP1 pentamers, and adjustment of their spacing and curvature on beads during VP1 coating, remain unclear but may be revealed by real-time atomic force microscopy.

Finally, coating of artificial materials with VP1 pentamers may improve their mono-dispersibility and stability. VP1-coated CMNPs were monodispersed in physiological and serum containing buffers, whereas uncoated CMNPs clustered and formed large multimeric structures [18]. Monodispersity of VP1-coated CMNPs enhanced the retention of these particles in body fluids of mice, as well as improving their targeting activity [18]. Moreover, SV40 VLPs retained their capsid structure even after incubation at 50 °C for 1 h in the presence of calcium chloride [24]. Therefore, VP1 coating may improve the structural stability of fragile materials such as liposomes, which are easily disrupted in body fluids. Thus, VP1 coating has potential for various applications in drug delivery.

## 5 Conclusion

This report showed that monomeric SV40 VP1 pentamers coat a variety of artificial beads, regardless of size and shape, due to their self-reassembly activity. VP1-coated materials may not only have a capsid-like surface but be mono-dispersible and stable, which may enhance their retention in body fluids and cell-targeting properties when used for drug delivery.

## Author contributions

M.K., K.D., H.F., Y.K., K.I., T.I., T.E., and M.H. performed the experiments. M.K., M.H., and H.H. conceived and designed the experiments. M.K., M.M., Y.Y., and H.H. wrote the manuscript. All authors discussed the results and commented on the manuscript.

## Additional information

The authors declare no competing financial interests. Correspondence and requests for materials should be addressed to H.H.

## References

[1] Liddington R.C., Yan Y., Moulai J., Sahli R., Benjamin T.L., Harrison S.C. (1991). Structure of simian virus 40 at 3.8-A resolution. Nature.

[2] Kosukegawa A., Arisaka F., Takayama M., Yajima H., Kaidow A., Handa H. (1996). Purification and characterization of virus-like particles and pentamers produced by the expression of SV40 capsid proteins in insect cells. Biochim. Biophys. Acta.

[3] Kawano M., Matsui M., Handa H. (2013). SV40 virus-like particles as an effective delivery system and its application to a vaccine carrier. Exp. Rev. Vaccines.

[4] Ishizu K.I., Watanabe H., Han S.I., Kanesashi S.N., Hoque M., Yajima H., Kataoka K., Handa H. (2001). Roles of disulfide linkage and calcium ion-mediated interactions in assembly and disassembly of virus-like particles composed of simian virus 40 VP1 capsid protein. J. Virol..

[5] Kanesashi S.N., Ishizu K., Kawano M.A., Han S.I., Tomita S., Watanabe H., Kataoka K., Handa H. (2003). Simian virus 40 VP1 capsid protein forms polymorphic assemblies in vitro. J. Gen. Virol..

[6] Kawano M.A., Inoue T., Tsukamoto H., Takaya T., Enomoto T., Takahashi R.U., Yokoyama N., Yamamoto N., Nakanishi A., Imai T., Wada T., Kataoka K., Handa H. (2006). The VP2/VP3 minor capsid protein of simian virus 40 promotes the in vitro assembly of the major capsid protein VP1 into particles. J. Biol. Chem..

[7] Enomoto T., Kukimoto I., Kawano M.A., Yamaguchi Y., Berk A.J., Handa H. (2011). In vitro reconstitution of SV40 particles that are composed of VP1/2/3 capsid proteins and nucleosomal DNA and direct efficient gene transfer. Virology.

[8] Tsukamoto H., Kawano M.A., Inoue T., Enomoto T., Takahashi R.U., Yokoyama N., Yamamoto N., Imai T., Kataoka K., Yamaguchi Y., Handa H. (2007). Evidence that SV40 VP1-DNA interactions contribute to the assembly of 40-nm spherical viral particles. Genes Cells.

[9] Inoue T., Kawano M.A., Takahashi R.U., Tsukamoto H., Enomoto T., Imai T., Kataoka K., Handa H. (2008). Engineering of SV40-based nano-capsules for delivery of heterologous proteins as fusions with the minor capsid proteins VP2/3. J. Biotechnol..

[10] Takahashi R.U., Kanesashi S.N., Inoue T., Enomoto T., Kawano M.A., Tsukamoto H., Takeshita F., Imai T., Ochiya T., Kataoka K., Yamaguchi Y., Handa H. (2008). Presentation of functional foreign peptides on the surface of SV40 virus-like particles. J. Biotechnol..

[11] Kitai Y., Fukuda H., Enomoto T., Asakawa Y., Suzuki T., Inouye S., Handa H. (2011). Cell selective targeting of a simian virus 40 virus-like particle conjugated to epidermal growth factor. J. Biotechnol..

[12] Cormode D.P., Jarzyna P.A., Mulder W.J., Fayad Z.A. (2010). Modified natural nanoparticles as contrast agents for medical imaging. Adv. Drug Deliv. Rev..

[13] Kawano M., Morikawa K., Suda T., Ohno N., Matsushita S., Akatsuka T., Handa H., Matsui M. (2014). Chimeric SV40 virus-like particles induce specific cytotoxicity and protective immunity against influenza A virus without the need of adjuvants. Virology.

[14] Kawano M., Matsui M., Handa H. (2014). SV40 virus-like particles as an effective delivery system and a vaccine platform. Virus-like Particles in Vaccine Development.

[15] Loo L., Guenther R.H., Basnayake V.R., Lommel S.A., Franzen S. (2006). Controlled encapsidation of gold nanoparticles by a viral protein shell. J. Am. Chem. Soc..

[16] Li F., Li K., Cui Z.Q., Zhang Z.P., Wei H.P., Gao D., Deng J.Y., Zhang X.E. (2010). Viral coat proteins as flexible nano-building-blocks for nanoparticle encapsulation. Small.

[17] Wang T., Zhang Z., Gao D., Li F., Wei H., Liang X., Cui Z., Zhang X.E. (2011). Encapsulation of gold nanoparticles by simian virus 40 capsids. Nanoscale.

[18] Enomoto T., Kawano M., Fukuda H., Sawada W., Inoue T., Haw K.C., Kita Y., Sakamoto S., Yamaguchi Y., Imai T., Hatakeyama M., Saito S., Sandhu A., Matsui M., Aoki I., Handa H. (2013). Viral protein-coating of magnetic nanoparticles using simian virus 40 VP1. J. Biotechnol..

[19] Salunke D.M., Caspar D.L., Garcea R.L. (1986). Self-assembly of purified polyomavirus capsid protein VP1. Cell.

[20] Garcea R.L., Salunke D.M., Caspar D.L. (1987). Site-directed mutation affecting polyomavirus capsid self-assembly in vitro. Nature.

[21] Yokoyama N., Kawano M.A., Tsukamoto H., Enomoto T., Inoue T., Takahashi R.U., Nakanishi A., Imai T., Wada T., Handa H. (2007). Mutational analysis of the carboxyl-terminal region of the SV40 major capsid protein VP1. J. Biochem..

[22] Caspar D.L., Klug A. (1962). Physical principles in the construction of regular viruses. Cold Spring Harb. Symp. Quant. Biol..

[23] Yan Y., Stehle T., Liddington R.C., Zhao H., Harrison S.C. (1996). Structure determination of simian virus 40 and murine polyomavirus by a combination of 30-fold and 5-fold electron-density averaging. Structure.

[24] Kawano M.A., Xing L., Tsukamoto H., Inoue T., Handa H., Cheng R.H. (2009). Calcium bridge triggers capsid disassembly in the cell entry process of simian virus 40. J. Biol. Chem..

